# A Simple Protocol for Using a LDH-Based Cytotoxicity Assay to Assess the Effects of Death and Growth Inhibition at the Same Time

**DOI:** 10.1371/journal.pone.0026908

**Published:** 2011-11-17

**Authors:** Shilo M. Smith, Michael B. Wunder, David A. Norris, Yiqun G. Shellman

**Affiliations:** 1 Department of Dermatology, School of Medicine, University of Colorado Denver, Aurora, Colorado, United States of America; 2 Department of Integrative Biology, University of Colorado, Denver, Colorado, United States of America; 3 Dermatology Section, Department of Veterans Affairs Medical Center, Denver, Colorado, United States of America; University of Saarland Medical School, Germany

## Abstract

Analyzing the effects on cell growth inhibition and/or cell death has been an important component of biological research. The MTS assay and LDH-based cytotoxicity assays are two of the most commonly used methods for this purpose. However, data here showed that MTS cell proliferation assay could not distinguish the effects of cell death or cell growth inhibition. In addition, the original LDH-based cytotoxicity protocol grossly underestimated the proportion of dead cells in conditions with growth inhibition. To overcome the limitation, we present here a simple modified LDH-based cytotoxicity protocol by adding additional condition-specific controls. This modified protocol thus can provide more accurate measurement of killing effects in addition to the measurement of overall effects, especially in conditions with growth inhibition. In summary, we present here a simple, modified cytotoxicity assay, which can determine the overall effects, percentage of cell killing and growth inhibition in one 96-well based assay. This is a viable option for primary screening for many laboratories, and could be adapted for high throughput screening.

## Introduction

Analyzing the effects on cell growth inhibition and/or cell death has been an important component of many biological research, especially in cancer treatment development. Accurately measuring the effects of treatments upon cells *in vitro* can be a challenging task. Proliferation assays like MTT [1], [2], [3] and MTS [4], [5] as well as ATP based [6], [7] assays are often used for primary screening. The MTS assay relies on the metabolism of the MTS reagent into formazan by dehydrogenase enzymes [4], [5], [8]. Proliferation assays like these have two major limitations: one they can give false positive results where specific aspects of cellular metabolism are affected [1], and two, they are unable to differentiate cell cycle inhibition and cellular death [9], [10]. To overcome these, Promega suggests multiplexing the assay with an LDH (lactate dehydrogenase) based cytotoxicity assay to allow for further quantification without having to repeat the setup and treatment phases. However, many therapeutic agents require multiple replication cycles for a killing effect to be evident, and dose dependent growth inhibition can act as a confounding variable when using a LDH-based cytotoxicity assay with current protocols, causing the amount of dead cells to be grossly underestimated [3], [9]. Here you will find a modified protocol for an assay that will allow for the more accurate determination of the total overall effect, the percentage growth inhibition as well as the percentage of killing all in one 96 well format assay that requires a simple absorbance plate reader and only one set of assay reagents.

This protocol is for use with Roche's cytotoxicity detection kit (catalog number 11 644 793 001). It allows one to use this kit to measure cellular death especially in experiments where relatively long treatment times are needed or significant cell cycle inhibition is present by adding condition specific controls. With this modified protocol, one can determine the percentage of cell death, overall effect as well as percentage of growth inhibition in one assay.

## Materials and Methods

### Cell lines and culture conditions

A375 cells were obtained from ATCC (American Type Culture Collection, Manassas, VA). Cells were maintained in RPMI 1640 medium (Sigma, St Louis MO) with 10% FBS (Gemini Bio-Products, West Sacremento, CA) and grown with 5% CO2 in an incubator at 37 degrees.

Cells were cultured in 96-well tissue culture plates at regular culture conditions with appropriate cell number in 100 µl media per well, and then being treated with bortezomib (LC laboratories, Woburn, MA) for 48 hours or PLX4720 (Selleck Chemicals, Houston, TX) for 72 hours before being subjected to indicated assays.

### Measurement of cell proliferation

The Cell Titer 96 Aqueous One solution cell proliferation assay (MTS assay; Promega Corp., Madison, WI) was used to quantify relative cell viability. Assays were performed according to the manufacturer's instructions.

### Measurement of cell cytotoxicity with the standard protocol

Cytotoxicity Detection Kit was purchased from Roche (Indianapolis, IN). TritonX-100 was purchased from Amersham Biosciences (Piscataway, NJ). The standard protocol assays reported here were performed according to the manufacturer's instructions.

### Measurement of cell cytotoxicity with the modified protocol

Two sets of replicates for each condition were used: one high control and one for the actual assay. Replicates of 100 µl of media without cells were used to serve as the blank for that condition. After 24 hours the cells and blanks were treated with 100 µl of their respective drug solutions at predetermined concentrations in serum free media.

On the day of the assay the cytotoxicty reagents were prepared according to the manufacturer's protocol. One non-sterile clean, clear, flat bottom 96-well plate was set aside and labeled as an assay plate for each experimental plate used. 4 µl (2% total volume) of triton X-100 was added to each of the high control wells, and mixed thoroughly using a multichannel pipette to ensure the cells membranes were properly degraded. The plate was then centrifuged for 5 min at 1000 RPM.

A multichannel was used to transfer 100 µl of supernatant from the top of all the wells of the experimental culture plate to the assay plate. Care was taken not to disturb the cells or draw up any debris. 100 µl of the mixed detection kit reagent was then added to each of the assay wells on top of the supernatant in rapid succession. The total volume in each well was 200 µl. The assay plates were then incubated at room temperature in the dark for twenty minutes. After which they were read using a standard plate reader with a reference wavelength of 490 nM.

### Statistical analysis

To determine whether the difference in cytotoxicity estimates for the two protocols was statistically significant, we bootstrapped the data to estimate 95% confidence intervals around the mean difference for each dosage level. We used a bootstrapping approach because we only had three to four trials at each dosage level, and thus could not reliably evaluate the assumption of normality needed to use a z- or t- based confidence interval (see R code in Supplemental materials, Text S1). Briefly, we randomly selected from among all condition assay wells, the media controls, and the condition-specific high controls before computing the percent cytotoxicity by the new protocol. This process was repeated 200 times for each protocol. We then computed the differences between each of the bootstrapped estimates for the modified and the standard protocols (modified-standard). To compute the 95% confidence interval, we sorted the estimates and excluded the lower and upper 2.5%. Thus, the lower bound of the intervals was computed by finding the 5^th^ lowest value and the upper limit by finding the 95^th^ lowest value. The 95% confidence intervals around the mean estimates for each protocol at each dosage were computed in the same way, but using only the protocol-specific estimates. That is, the 95% confidence intervals that appear in the figures were computed by sorting the bootstrapped estimates and excluding the upper and lower 2.5% of values. For each dose level, if the 95% confidence interval around the mean difference between the protocol estimates for cytotoxicity did not include 0, then the difference in cytotoxicity estimates was statistically significant at p≤0.05. Likewise, whenever the 95% confidence interval around the estimated % cytotoxicity for one protocol does not contain the mean % cytotoxicity for the other protocol at the same dose level, the differences are statistically significant at p≤0.05.

## Results and Discussion

**Figure 1:**
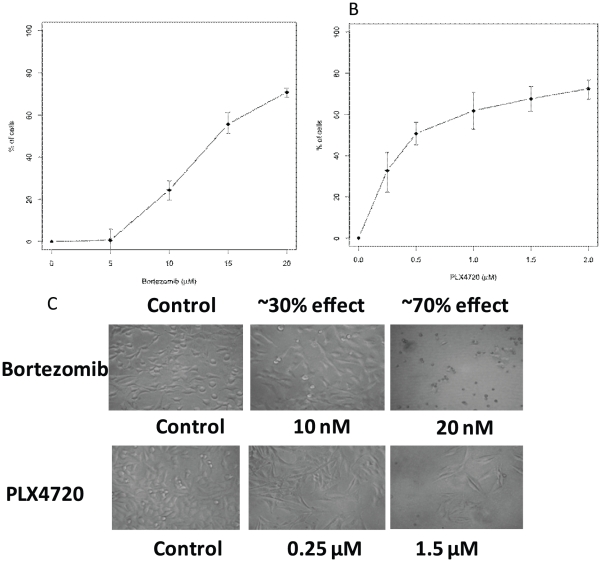
MTS cell proliferation assay could not distinguish the effects of cell death or cell growth inhibition. MTS assays were performed on A375 cells that were treated with bortezomib (A) or PLX4720 (B). The Y-axis is the % of cells affected by the indicated treatment compared to the total cells treated with vehicle control DMSO. The error bars are the estimated 95% confidence interval around the mean (see Statistical analysis in Materials and Method section). C. Images of cells from the experiments performed for A and B. Results here are representative of 3 independent experiments.

While there are a variety of assays available to determine the effects of a specific condition, historically tetrazolium salt (XTT, MTT, MTS) and ATP based assays have been used as a method of performing preliminary screenings involving death and proliferation studies[1], [4], [5], [8], [9], [11], [12], [13]. It has long been known that MTS and ATP based assays may overestimate the effectiveness of a drug that alters cell metabolism but not cell viability due to the fact that the assays measure cellular metabolism as indirect readout for cell proliferation[1]. In addition, our data also shows that these assays failed to differentiate between cell death and growth inhibition (Figure 1). The curves in Figure 1A and 1B have points representing approximately 30% and 70% effectiveness on them as measured by MTS assays. Additional morphological examination of these conditions via light microscopy indicated that many of the cells in 20 nM bortezomib were dead, but the vast majority of the cells in 1.5 µM PLX4720 were alive (Figure 1C). Thus, the bortezomib had killing effects on these cells while PLX4720 had cell-growth-inhibition effects. However, the death and growth inhibition were indistinguishable from the graphs in Figure 1A and 1B demonstrating that MTS assays cannot distinguish the effects of cell growth inhibition and cell killing. Similar results have been observed in multiple cell lines and multiple treatments (data not shown).

**Figure 2:**
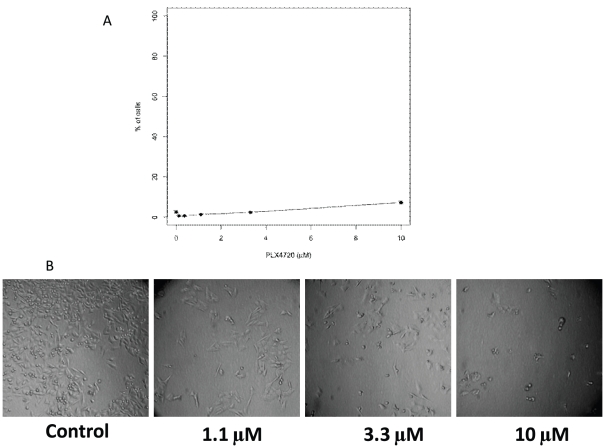
Original cytotoxicity protocol grossly underestimated the proportion of dead cells in conditions with growth inhibition. A375 cells were treated with PLX4720 then assayed using standard protocol with Cytotoxicity Detection Kit (A). The Y-axis is the % of cells killed by the indicated treatment compared to the total cells treated with vehicle control DMSO. The error bars are the estimated 95% confidence interval around the mean (see Statistical analysis in Materials and Method section). (B) Images of cells from the experiments performed for A.

LDH and G6PD based assays can be used to determine the percentage of dead cells under a given condition [3], [9]. However, in the presence of growth inhibition, such as we see with PLX4720 treatment, the LDH based assay can grossly underestimate the percentage of dead cells (Figure 2). This is because the standard protocol bases the total possible LDH release on a single control where Triton X-100 is added to the culture to cause the breakdown of all of the cells. This is done on the vehicle treated condition. The supernatant from these wells is then used as a reference for the total possible amount of LDH. LDH based assays are only able to detect cells that are in a late enough stage of demise to release LDH. In cancer research, as in other areas of research, there are many compounds that require treatment times in excess of 48 hours to induce death. Herein lies a problem with the original protocol; many of these compounds also induce cell cycle arrest. Thus as time passes the number of replication cycles in the control group becomes increasingly higher than that of the treated groups, the total number of cells and as a result the total LDH possible now varies in a dose dependant manner.

**Figure 3:**
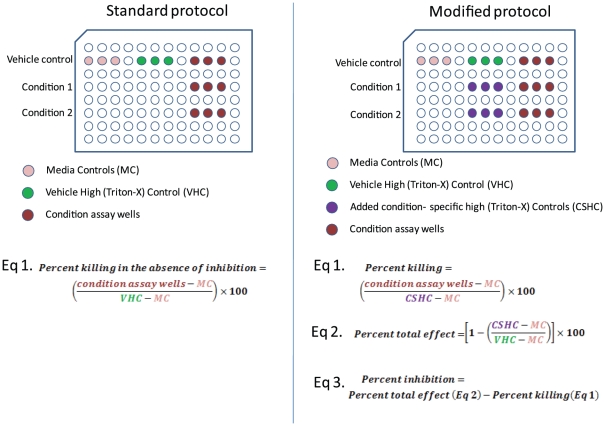
Comparison of experimental layout between the standard and modified protocols. Additional controls in the modified protocol increased the accuracy as well as the amount of data obtainable from one assay. The added controls are shown in purple on a sample layout of the modified protocol. The three equations used to analyze the data all utilize the added controls.

Figure 2 illustrates this scenario: At 10 µM, a significant percentage of the cells appear dead by morphological examination, yet the cytotoxicity assay detects less than 10% death in that condition. This is due to cell-growth inhibition effects of PLX4720 (Figure 2B). Using the standard protocol, the total LDH possible was based on the controls for the DMSO control, which had far more cells thus containing higher amounts of total LDH than that in 10 µM treated conditions. To overcome this problem, one easy way is to modify the standard protocol and use condition-specific-controls for each condition by adding samples to be lysed by Triton X-100 as the high control for each condition. This would allow for more accurate calculation of the percentage of cells killed in conditions even when significant cell growth inhibition was present. Figure 3 illustrates the differences between the modified protocol and the standard protocol.

**Figure 4:**
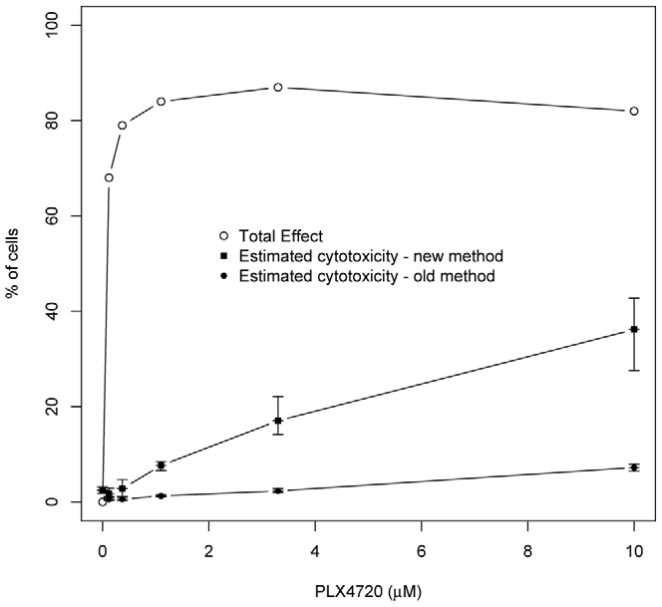
Modified cytotoxicity protocol gave measurements of overall effects on the cells and more accurate measurement of percentage of dead cells in conditions with growth inhibition. The experiment from Figure 2 was re-calculated using all of the controls for the modified protocol. The total LDH from the control wells is also plotted to show the overall effect of that condition. The same plot of Figure 2 from old method is also presented here for comparison. The error bars are the estimated 95% confidence interval around the mean (see Statistical analysis in Materials and Method section). Whenever the 95% confidence interval around the estimated % cytotoxicity for one protocol does not contain the mean % cytotoxicity for the other protocol at the same dose level, the differences are statistically significant at p≤0.05.

As a result of using condition-specific-controls in the calculations, the assay now indicates that 36% of these cells are dead (Figure 4); a number that corresponds more closely with what is visibly evident via light microscopy (Figure 2B). The mean differences in estimated cytotoxicity and associated 95% confidence intervals for each dose level are reported in Table 1. Results indicated that there were dramatic differences in estimates of cytotoxicity between the old and new methods, statistical analysis also confirmed that significant differences between these two methods, especially for the higher doses.

**Table 1:** Mean differences and 95% CI around the mean difference in estimated % cytotoxicity for the two protocols (modified protocol – standard protocol) at each dose level.

Dose (µM)	Mean Difference (%)	Lower Limit 95% CI	Upper Limit 95% CI
**0.00**	−0.06	−0.09	−0.03
**0.12**	1.15	0.77	2.04
**0.37**	2.21	0.86	3.69
**1.10**	6.35	5.54	6.99
**3.30**	14.65	12.17	19.34
**10.00**	29.01	21.00	35.51

Note: For each dose level, if the 95% confidence interval around the mean difference between the protocol estimates for cytotoxicity did not include 0, then the difference in cytotoxicity estimates was statistically significant at p≤0.05.

The total LDH controls can also be used to calculate the total effect of a specific condition (Figure 3). If you were to subtract the percent dead via the new method from the total affect you will have the percentage of effective growth inhibition. Therefore, we find that 46% of the effect in the 10 µM condition was growth inhibition. Thus with one assay you can determine the percent of inhibition, the percentage of cells killed and the total overall effect of a drug or condition.

Table 2 compares the various commonly used, inexpensive, primary screening methods that can be used for high throughput screening. Our modified method is the only one has the capacity to be quantitative, be able to distinguish cell death versus growth inhibition without need of expensive specialized equipment.

**Table 2:** Comparison of inexpensive, commonly used primary screens.

Assay	Quantitative	High throughput friendly)	Death VS inhibition	Specialized equipment required
MTS, MTT, XTT	Yes	Yes	No	No
ATP based (glow)	Yes	Yes	No	No
Cytotoxicity	if no growth inhibition or short time point	Yes	No	No
ModifiedCytotoxicity	Yes	Yes	Yes	No

In summary, we present here a simple, modified cytotoxicity assay that can determine the overall effects, percentage of cell killing and growth inhibition in one 96-well based assay. While the protocol is designed for a 96-well plate it could also easily be modified to other plate styles for high throughput screening in applications like drug discovery.

## Supporting Information

Text S1 **R code used for statistical analysis.**
(DOCX)
