# Supplementary material for: A Simple Protocol for Using a LDH-Based Cytotoxicity Assay to Assess the Effects of Death and Growth Inhibition at the Same Time
Source: PLoS One. 2011 Nov 17;6(11):e26908. doi: 10.1371/journal.pone.0026908 (PMC3219643; doi:10.1371/journal.pone.0026908)
Supplement: Text S1 — R code used for statistical analysis. (DOCX) [file pone.0026908.s001.docx]

**Supplemental materials**

**# this code is for bootstrapping experimental results presented in Smith et al.**

# M.B. Wunder, September 2010; modified September 2011

# read in the data

x=data.frame(dose=factor(c(0,0,0,0.12,0.12,0.12,0.37,0.37,0.37,1.1,1.1,1.1,3.3,3.3,3.3,10,10,10)),cshc=c(1.936,1.709,1.724,0.823,0.636,0.619,0.513,0.512,0.541,0.447,0.445,0.424,0.427,0.376,0.407,0.472,0.481,0.542),caw=c(0.22,0.204,0.219,0.18,0.18,0.187,0.188,0.189,0.198,0.197,0.199,0.202,0.222,0.221,0.23,0.298,0.315,0.31),mc=c(0.176,0.179,0.173,0.1735,0.175,0.172,0.1825,0.184,0.181,0.1795,0.181,0.178,0.1885,0.19,0.187,0.2015,0.205,0.198))

# these are the experimental data - cshc is the condition-specific high control, caw is condition assay well, mc is the media control. The Vehicle high control is represented by the values for csch that are associated with dose level 0

f1a=data.frame(dose=factor(c(0,0,0,0,5,5,5,5,10,10,10,10,15,15,15,15,20,20,20,20)),bodval=c(0.289,0.297,0.309,0.334,0.307,0.298,0.299,0.317,0.24,0.228,0.236,0.224,0.12,0.147,0.132,0.148,0.096,0.087,0.093,0.082))

# these are the data for figure 1a

f1b=data.frame(dose=factor(c(0,0,0,0,0.25,0.25,0.25,0.25,0.5,0.5,0.5,0.5,1,1,1,1,1.5,1.5,1.5,1.5,2,2,2,2)),bodval=c(0.42225,0.39725,0.37525,0.49825,0.24425,0.30725,0.26725,0.31625,0.21825,0.20425,0.20625,0.20425,0.12425,0.17725,0.14625,0.20525,0.12325,0.11325,0.15625,0.15625,0.10925,0.12825,0.09625,0.13125))

# these are the data for figure 1b

# setup routine to bootstrap 95% CI for figure 1 data

# initialize some objects

reps = 200; adat=NULL;bdat=NULL

d=length(levels(f1a$dose))

# bootstrap routine for fig 1a

da=f1a[f1a$dose==0,]

a=sample(da[,2],4*reps,replace=T)

za=matrix(a,nrow=reps,ncol=4)

z1m=rowMeans(za)

adat=c(0,0,0)

for (dl in 2:d){

da=f1a[f1a$dose==levels(f1a$dose)[dl],]

a=sample(da[,2],4*reps,replace=T)

za=matrix(a,nrow=reps,ncol=4)

zam=100-(rowMeans(za)/z1m*100)

adat=c(adat,mean(zam),zam[order(zam)[5]],zam[order(zam)[195]])

}

aplot=matrix(adat,nrow=d,ncol=3,byrow=T)

# bootstrap for fig 1b

d=length(levels(f1b$dose))

db=f1b[f1b$dose==0,]

b=sample(db[,2],4*reps,replace=T)

zb=matrix(b,nrow=reps,ncol=4)

z1m=rowMeans(zb)

bdat=c(0,0,0)

d=length(levels(f1b$dose))

for (dl in 2:d){

db=f1b[f1b$dose==levels(f1b$dose)[dl],]

b=sample(db[,2],4*reps,replace=T)

zb=matrix(b,nrow=reps,ncol=4)

zbm=100-(rowMeans(zb)/z1m*100)

bdat=c(bdat,mean(zbm),zbm[order(zbm)[5]],zbm[order(zbm)[195]])

}

bplot=matrix(bdat,nrow=d,ncol=3,byrow=T)

figa.data=data.frame(dose=c(0,5,10,15,20),mu=aplot[,1],lcl=aplot[,2],ucl=aplot[,3])

figb.data=data.frame(dose=c(0,0.25,0.5,1,1.5,2),mu=bplot[,1],lcl=bplot[,2],ucl=bplot[,3])

# fig 1a

plot(figa.data$dose,figa.data$mu,pch=16,type="b",xlab=expression(paste("Bortezomib (",mu,"M)")),ylab="% of cells",ylim=c(0,100))

arrows(figa.data$dose,figa.data$mu,figa.data$dose,figa.data$ucl,length=0.05,angle=90,lty=1,code=3)

arrows(figa.data$dose,figa.data$mu,figa.data$dose,figa.data$lcl,length=0.05,angle=90,lty=1,code=3)

# fig 1b

plot(figb.data$dose,figb.data$mu,pch=16,type="b",xlab=expression(paste("PLX4720 (",mu,"M)")),ylab="% of cells",ylim=c(0,100))

arrows(figb.data$dose,figb.data$mu,figb.data$dose,figb.data$ucl,length=0.05,angle=90,lty=1,code=3)

arrows(figb.data$dose,figb.data$mu,figb.data$dose,figb.data$lcl,length=0.05,angle=90,lty=1,code=3)

# cleanup

rm(reps,d,adat,bdat,da,a,za,z1m,dl,zam,zbm,aplot,db,b,zb,bdat,bplot)

# setup routine for the experimental data

# initialize some objects

reps = 200;

d=length(levels(x$dose)); p.dif=NULL;d.dif=NULL

dosr.n=NULL; dosr.o=NULL; dosx.n=NULL; dosx.o=NULL

# bootstrap routine

for (dl in 1:d){

dat=x[x$dose==levels(x$dose)[dl],]

r.n=NULL; r.o=NULL; d.p=NULL

for (i in 1:reps){

a=sample(1:3,3,replace=F)

rhon=(dat[a[1],3]-dat[a[3],4])/(dat[a[2],2]-dat[a[3],4])

r.n=c(r.n,rhon)

rho=(dat[a[1],3]-dat[a[3],4])/(x[a[2],2]-dat[a[3],3])

r.o=c(r.o,rho)

d.p=c(d.p,rhon-rho)

}

dosr.n=c(mean(r.n),r.n[order(r.n)[5]],r.n[order(r.n)[195]])

dosx.n=c(dosx.n,dosr.n)

dosr.o=c(mean(r.o),r.o[order(r.o)[5]],r.o[order(r.o)[195]])

dosx.o=c(dosx.o,dosr.o)

p.dif=c(mean(d.p),d.p[order(d.p)[5]],d.p[order(d.p)[195]])

d.dif=c(d.dif,p.dif)

}

# collect results

n=matrix(dosx.n,nrow=d,ncol=3,byrow=T)

o=matrix(dosx.o,nrow=d,ncol=3,byrow=T)

dose.diff=matrix(d.dif*100,nrow=d,ncol=3,byrow=T)

fig.data=data.frame(dose=c(0,0.12,0.37,1.1,3.3,10.0),mu.n=n[,1]*100,lcl.n=n[,2]*100,ucl.n=n[,3]*100,mu.o=o[,1]*100,lcl.o=o[,2]*100,ucl.o=o[,3]*100,effect=c(0,68,79,84,87,82))

# fig 2

plot(fig.data$dose,fig.data$mu.o,pch=16,type="b",xlab=expression(paste("PLX4720 (",mu,"M)")),ylab="% of cells",ylim=c(0,100))

arrows(fig.data$dose,fig.data$mu.o,fig.data$dose,fig.data$ucl.o,length=0.05,angle=90,lty=1,code=3)

arrows(fig.data$dose,fig.data$mu.o,fig.data$dose,fig.data$lcl.o,length=0.05,angle=90,lty=1,code=3)

# fig 4

plot(fig.data$dose,fig.data$effect,type="b",xlab=expression(paste("PLX4720 (",mu,"M)")),ylab="% of cells",ylim=c(0,100))

points(fig.data$dose,fig.data$mu.n,type="b",pch=15)

points(fig.data$dose,fig.data$mu.o,type="b",pch=16)

arrows(fig.data$dose,fig.data$mu.n,fig.data$dose,fig.data$ucl.n,length=0.05,angle=90,lty=1,code=3)

arrows(fig.data$dose,fig.data$mu.n,fig.data$dose,fig.data$lcl.n,length=0.05,angle=90,lty=1,code=3)

arrows(fig.data$dose,fig.data$mu.o,fig.data$dose,fig.data$ucl.o,length=0.05,angle=90,lty=1,code=3)

arrows(fig.data$dose,fig.data$mu.o,fig.data$dose,fig.data$lcl.o,length=0.05,angle=90,lty=1,code=3)

legend(2,60,c("Total Effect","Estimated cytotoxicity - new method","Estimated cytotoxicity - old method"),pch=c(1,15,16),bty="n")

# clean up

rm(reps,d,dl,dat,r.n,r.o,a,rho,dosr.n,dosr.o,dosx.n,dosx.o,n,o,d.p,p.dif,d.dif)
